# Altered circular RNA expressions in extracellular vesicles from bronchoalveolar lavage fluids in mice after bacterial infections

**DOI:** 10.3389/fimmu.2024.1354676

**Published:** 2024-04-04

**Authors:** Heedoo Lee, Rokgi Hong, Yang Jin

**Affiliations:** ^1^ Division of Pulmonary and Critical Care Medicine, Department of Medicine, Boston University, Boston, MA, United States; ^2^ Department of Biology and Chemistry, Changwon National University, Changwon, Republic of Korea

**Keywords:** macrophage activation, inflammation, bacterial infection, circular RNA, endotoxin, extracellular vesicles (EVs). LPS. bronchoalveolar lavage fluid (BALF)

## Abstract

Circular RNAs (circRNAs) are a class of transcripts that often are generated by back-splicing that covalently connects the 3′end of the exon to the 5′end. CircRNAs are more resistant to nuclease and more stable than their linear counterparts. One of the well-recognized roles of circRNAs is the miRNA sponging effects that potentially lead to the regulation of downstream proteins. Despite that circRNAs have been reported to be involved in a wide range of human diseases, including cancers, cardiovascular, and neurological diseases, they have not been studied in inflammatory lung responses. Here, we analyzed the circRNA profiles detected in extracellular vesicles (EVs) obtained from the broncho-alveolar lavage fluids (BALF) in response to LPS or acid instillation in mice. Next, we validated two specific circRNAs in the BALF-EVs and BALF cells in response to endotoxin by RT-qPCR, using specific primers targeting the circular form of RNAs rather than the linear host RNAs. The expression of these selected circRNAs in the BALF inflammatory cells, alveolar macrophages (AMs), neutrophils, and lung tissue were analyzed. We further predicted the potential miRNAs that interact with these circRNAs. Our study is the first report to show that circRNAs are detectable in BALF EVs obtained from mice. The EV-cargo circRNAs are significantly altered by the noxious stimuli. The circRNAs identified using microarrays may be validated by RT-qPCR using primers specific to the circular but not the linear form. Future studies to investigate circRNA expression and function including miRNA sponging in lung inflammation potentially uncover novel strategies to develop diagnostic/therapeutic targets.

## 1 Introduction

Circular RNAs (circRNAs) refer to a group of newly discovered, single-stranded coding or non-coding RNAs. Compared to the traditional linear mRNAs, circRNAs contain a closed-loop structure without free ends. They are often generated by back-splicing of mRNA precursors (pre-miRNAs) and thus forming a unique circular structure via the covalently closed continuous loop (1–5). There are no distinct 3’ and 5’ ends in circular RNAs as they have been joined together. This feature renders them more resistant to exonuclease-mediated degradation than their linear RNA counterparts. This structure is also more conserved among different species. Many reports have shown that the half-life of circRNAs can be 2-3 folds longer (1–5).

CircRNA was initially discovered and reported in 1976 (6), they were believed as junk products resulting from mRNA mis-splicing. Over the past three decades, circRNAs have been considered a type of non-coding RNA. Recent evidence demonstrates that circRNAs are present in many cell types in humans. Subsequently, the functions of circRNAs draw much more attention. Accumulating evidence shows that some circRNAs code for proteins (7, 8). More recognized functions of circRNAs include gene regulations and microRNA (miRNA) sponging (9). Despite the scatted reports, the biological functions of most circRNAs remain unexplored.

Circular RNA has been linked to some diseases such as cancer (10), diabetes (11), cardiovascular diseases (12), age-related diseases (13), osteoarthritis (14), stress (15), and viral diseases (16, 17). Due to the lack of 5’ or 3’ ends and resistance to exonuclease-mediated degradation, circRNAs are presumably more stable than most linear RNA in cells, tissues, and body fluids (1–5). Their half-lives can be longer than 48 hours (1–5, 18) and are easily detected using real-time PCR (19, 20). Therefore, the identification of circRNAs provides a potential strategy for the development of novel diagnostic and prognostic biomarkers for human diseases. Moreover, circRNAs are highly abundant in blood (21) and detectable in urine samples (22).

CircRNAs can be secreted into the extracellular spaces and thus detectable in body fluids. Given that circRNAs are more resistant to exonuclease and more difficult to degrade, secretion from the cells may be one of the regulatory methods to control the intracellular level of circRNAs and/or clear them from the cell. Extracellular vesicle (EV)-mediated release of circRNAs may be one of the tightly regulatory steps. Indeed, recent studies have shown that circRNAs are enriched in extracellular vesicles (EVs) and secreted in an EV-mediated manner. EVs are spherical bilayer vesicles released by cells into extracellular spaces. EVs mediate intercellular cross-talks, largely via delivering the EV cargoes into the recipient cells. Among the components of EVs, circRNAs are a novel type of noncoding RNA (23). Li et al. first identified more than 1000 circRNAs in human serum exosomes and revealed that circRNAs were enriched in exosomes of liver cancer cells using RNA sequencing (RNA‐seq) analyses (23). Currently, very little is known about the metabolism of circRNAs within the cell. Presumably, considering their high degree of stability, circRNAs may accumulate intracellularly and may be toxic. One hypothesis is that cells cope with circRNA accumulations via EV-mediated secretion. The evidence for this hypothesis is that circRNAs are easily detected in EVs and are enriched in EVs compared to their intracellular levels relative to linear forms of the same genes.

Despite the emerging reports on the involvement of circRNAs and human diseases, very few studies focus on non-cancer lung diseases, e.g., inflammatory lung responses to noxious stimuli such as bacterial infections, toxic chemical exposure, and hyperoxia exposure. Alveolar macrophages form the first line of defense against pollutants and pathogenic microbes and initiate an innate immune response in the lung. Following noxious stimuli, bone marrow-derived monocytes are recruited to the lung and differentiate into alveolar macrophages (24–26). Alveolar macrophages are involved in recruiting neutrophils to the site of infection and carry multiple functional roles including but not limited to host defense, recognition of pathogens, clearance of pathogen and debris, initiation and resolution of lung inflammation, and repair of damaged tissue (27). Under physiological conditions, alveolar macrophages produce low levels of inflammatory cytokines, maintain high phagocytic activity, and generally suppress inflammation and adaptive immunity (28). Alveolar macrophages (AMs), along with all other lung cells, secrete and excrete molecules/cytokines in the alveolar sac. Bronchoalveolar lavage fluid (BALF) contains the immune cells in lung alveoli and can, provide crucial information about the immunological response and host defense occurring in the lungs. To the best of our knowledge, circRNA levels and profiles in BALF have not been explored, particularly in the setting of inflammatory lung responses.

In this report, we initially studied the profile of circRNAs in BALF-EVs, in response to both sterile and infectious stimuli. Next, we confirmed several specific circRNAs using RT-qPCR. We evaluated the expressions of these circRNAs in EVs from BALF and serum, in inflammatory cells, and in lung tissue. Our study first reported a potentially important regulator in macrophage activation in response to bacterial infection in the lungs and may open a novel insight into the host defense and innate immunity in the lungs.

## 2 Materials and methods

### 2.1 Cells, cell culture, reagents, and supplies

Cell culture media including RPMI-1640 and Dulbecco’s Modified Eagle’s Medium (DMEM), phosphate-buffered saline (PBS), fetal bovine serum (FBS), and Penicillin Streptomycin (Pen Strep) were purchased from Thermo-Fisher Scientific (Cambridge, MA). Alveolar macrophages were isolated from wild-type (WT) C57BL/6 mice at 8-12 weeks of age (Charles River, Wilmington, MA) as previously described (29). Mouse lung alveolar type I cell line (E-10) cells were obtained from Dr. Joseph Mizgerd (Pulmonary Center, Boston University). MHS cells (CRL-2019, ATCC) were cultured in RPMI-1640 with 10% FBS and 1% Pen Strep. All cells were maintained in a 37°C incubator with 5% CO2.

### 2.2 Mice

Wild type (WT) C57BL/6 mice (male and female), 8 weeks of age, weight approximately 20 grams, were obtained from the (Charles River, Wilmington, MA). All protocols and methods involving animals were approved by the Institutional Animal Care and Use Committee of Boston University, following approved guidelines.

### 2.3 Animal study

Mice were anesthetized with 1 ml of isoflurane for 10-20 seconds, and then either LPS (1 µg per mouse) or HCl (0.1 N, 50 µl per mouse) was intratracheally instilled (i.t.) into the lungs.

At 24h after administration, BALFs were collected from the mouse lungs, and lung tissues that were not subjected to the BALF collection were used for histology and isolation of lung tissue inflammatory cells. For BALF collection, mice were euthanized using carbon dioxide (CO_2_). A 20-gauge catheter was inserted into the trachea, and PBS (1ml) was injected into the lungs. Subsequently, BALF was collected from the lungs.

#### 2.3.1 BAL and inflammatory cells

To induce lung inflammation, WT mice were anesthetized and intratracheally instilled with LPS as above. After 24h, mice were euthanized and the BALF was collected. BALF was cytocentrifuged at 300 g for 5 minutes to collect BALF cells.

#### 2.3.2 Isolation of mouse alveolar macrophages

Mouse alveolar macrophages (AMs) were obtained as previously reported (29). Briefly,

BALF cells were obtained as described above and resuspended in 100µl of flow/sorting buffer. Next, we block the nonspecific antibody binding to the Fc receptor by adding a mouse Fc block. We performed antibody staining along with fluorescence minus one (FMO) and single-stain controls. We evaluated the BAL cells with a cell analyzer followed by single-cell flow cytometry analysis by the appropriate analysis software. AMs were isolated by a fluorescence-activated cell sorter directly into 1 mL mouse AM culture medium when AMs are intended for *in vitro* culture or *in vivo* cell transfer. Alternatively, sort AMs into 1 mL Lysis buffer supplemented with 10 μL/mL β-Mercaptoethanol for RNA extraction.

### 2.4 Extracellular vesicle and exosome isolation and characterization

Exosomes were isolated from mouse bronchoalveolar lavage fluid (BALF). To obtain BALF, a total of 2 mL of PBS was used for lung lavage as previously described (30). BALF was centrifuged at 300g for 10 minutes to remove cells, followed by 2,000g for 20 minutes to separate apoptotic bodies, and 16,000g for 40 minutes to separate microvesicles (30). The resulting supernatants were then ultracentrifuged at 100,000g for 1 hour to obtain exosomes (30). TEM images were used to examine the EV morphology by the Experimental Pathology Laboratory Core, Boston University School of Medicine.

### 2.5 RNA isolation, reverse transcription, RT-qPCR, and circRNA profiling/arrays

RNeasy Plus Mini Kits (QIAGEN, Valencia, CA) were used for the purification of total RNA from cells. Single-stranded cDNAs were prepared using a High-Capacity cDNA Reverse Transcription Kit (Thermo Fisher Scientific, Waltham, MA). PowerUp™ SYBR™ Green Master Mix (Applied Biosystems, Foster City, CA) and QuantStudio 3 system (Applied Biosystems, Foster City, CA) were used for real-time quantitative PCR (qPCR). The list of primers is shown in the results and figures. For the circRNA profiling using microarrays, we used the service from Arraystar (Rockville, MD). Briefly, a total of 14236 transcript-specific probes for mouse circRNAs were used—random primer labeling coupled with RNase R sample pretreatment to ensure specific and efficient labeling of circular RNAs.

### 2.6 Statistics

Data are presented as mean ± SD. Comparisons between groups were analyzed using a two-tailed unpaired Student’s *t-*test. Values with *P <0.05* were considered as significant.

## 3 Results

### 3.1 Significantly altered circRNA profiles in EVs separated from BALF after LPS or HCL intra-tracheal instillation

**Figure 1:**
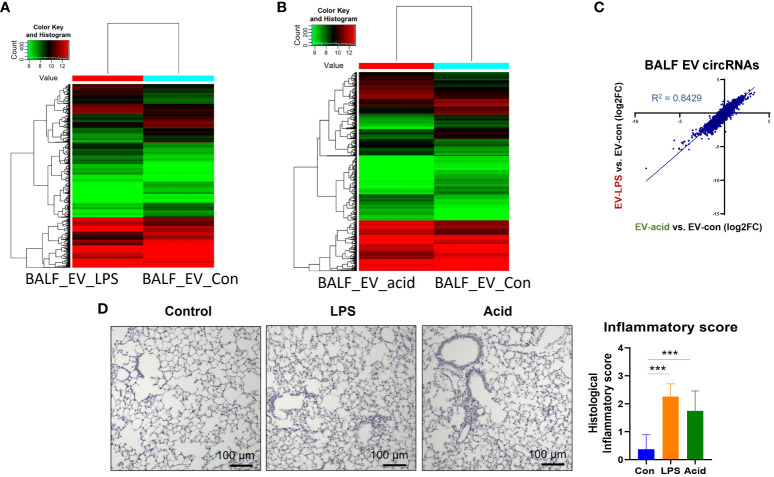
CircRNA profiles in BALF EVs, serum EVs and BALF cells. Three mice were used in this experiment. BALF and serum were collected and pooled after exposure to PBS or LPS (1µg/mouse) or HCl (0.1N) for 24h. EVs were separated using ultracentrifugation as previously described (30). RNA was isolated from the EVs and subjected to circRNA microarrays by Arraystar Inc (Rockville, MD). Heatmap of the circRNA arrays in EVs obtained from BALF in the absence and presence of noxious stimuli. **(A)** BALF EVs obtained from mice that were exposed to PBS or LPS. **(B)** BALF EVs obtained from mice that were exposed to PBS or HCL. **(C)** the changes of EV-cargo circRNA profiles between non-infectious stimuli (HCL) and endotoxin (LPS) were expressed in Log2 fold change. **(D)** A representative picture showing the histological changes was presented here. The inflammatory score was determined by a double-blinded examiner using five lung sections per mouse, and three mice per treatment. The mean value is graphed and presented. ***p<0.05.

We initially examined the circRNA profiles in BALF EVs obtained from mice in the absence and presence of LPS or HCL intra-tracheal instillation. WT C57BL/J mice (8 weeks, 20gm) were exposed to LPS (1 µg per mouse) or HCl (0.1 N, 50 µl per mouse) as described in the Materials and Methods. HCL *i.t.* and LPS *i.t.* are common mouse models to mimic the condition of exposure to sterile and infectious stimuli, i.e., acid aspiration or G-bacterial pneumonia (30). After 24h, BALF was obtained and EVs were separated as previously described (30). BALF EVs from 3 mice were pooled together and subjected to circRNA profiling using the microArrays by Arraystar (Rockville, MD). As shown in **Figure 1**, EV-cargo circRNA profiles were altered after acid instillation and LPS instillation in BALF (**Figures 1A, B**). This alteration was presented using a plot with a logarithmic scale (**Figure 1C**). A robust alteration of the circRNA profiles in EVs obtained from BALF was observed after both stimuli. Under the same condition, the lung histology and inflammation scores were measured, and a representative picture was shown in **Figure 1D**.

### 3.2 Confirmation of a specific circRNA level in the BALF EVs obtained from mice exposed to sterile or infectious stimuli

**Figure 2:**
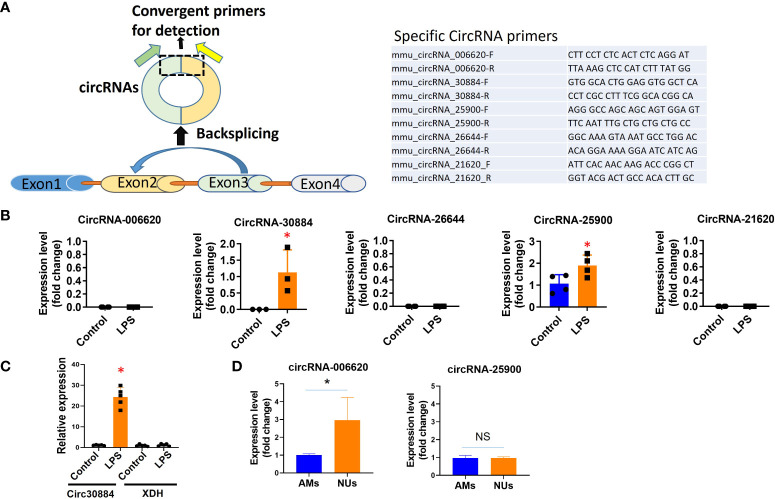
Validation of individual circRNAs detected in microarray profiling using RT-qPCR. WT mice were exposed to Acid (0.1 N HCl) or LPS (1µg) for 1 day. BALF EVs and cells were collected and circRNAs were evaluated using qPCR (normalized to β-actin or GAPDH). **(A)** To validate the specific circRNAs using RT-qPCR, we first designed the specific primers targeting the BSJ region to detect circular forms of RNAs, as illustrated here (left panel). The specific sequence of each specific circRNAs is listed on the right panel. **(B)** Validation of specific circRNAs using RT-qPCR in BALF EVs. We selected five circRNAs that were highly altered in the BALF EVs using microarray profiling. Next, we validated the expression and alteration of each circRNA using RT-qPCR in BALF EVs. *p<0.05. **(C)** To confirm that the specific primers used to detect circRNAs fail to detect the linear form host RNA. Using circ30884 as an example, we confirmed that using the primer listed above, we failed to detect its host linear RNA XDH. The figures shown here represent repeats from three independent experiments. *p<0.05. **(D)** AMs and neutrophils were separately isolated from LPS-exposed mouse lung as the above. Circular RNAs were then detected using RT-qPCR. *p<0.05, ns, not significant.

To validate the results from microarray profiling, after comparing them with the published human circRNA sequences, we selected several circRNAs with great folds of difference and conserved in both mice and humans. We used the real-time RT-qPCR to validate the above observation in 4.1. As illustrated in **Figure 2A**, circRNAs are often generated by back splicing. We designed the specific primers for RT-qPCR (**Figure 2A** right panel) accordingly. Our primary interests fall into the inflammatory lung responses after pneumonia. Therefore, we focus on the effects of LPS on BALF-EVs here.

The level of expression of the selected circRNAs in BALF-EVs was first confirmed using RT-qPCR. Compared to the profiling data, circRNA 30884 and 25900 were detected in BALF EVs, but not circ006620, 26644, and 21620 (**Figure 2B**). One of the key factors in the circRNA research is to ensure the primers are specific to the circular form of RNAs but not its linear form. To ensure that the host gene was not detected but only in the circular form using the designated primers, we also analyzed the host gene using the same samples and primers by RT-qPCR. Using circ30884 as an example, no changes were found for host genes of circ30884 using the circRNA-specific primers (**Figure 2C**), suggesting that the primers are specific to the circular form.

### 3.3 Analysis of the selected circRNAs in lung tissue and potential origin cells

The majority of the BALF EVs originated from lung cells or immune cells, depending on the type of stimulation (30). We evaluated the expressions of the selected circRNAs in lung tissue, epithelial cells, alveolar macrophages (AMs), and neutrophils. A differential expression of circ006620 and circ25900 was observed between AMs and neutrophils (**Figure 2D**), suggesting that each circRNA should be evaluated in the individual type of cells. Note that we could not compare the circRNA levels in untreated neutrophils and LPS-treated neutrophils, as the amount of neutrophil in BALF without LPS was too low. As shown in the **Supplementary Figure 1**, the circ25900 and circ006620 were evaluated in lung tissue and alveolar epithelial cells. Differential expression and induction after LPS were observed. Using immortalized AMs, we next performed a time course of circ25900 expression after LPS. We found that LPS-induced circ25900 attenuated after prolonged treatment (**Supplementary Figure 1B** lower panel), suggesting for this specific circRNA, LPS-induced upregulation occurred at the early phase of exposure.

The information from **Figure 2**; **Supplementary Figure 1** confirmed that RT-qPCR can be used to validate that circRNA screening obtained by microarrays. A time course of expression may be performed to delineate the LPS-mediated induction. Differentially expressed circRNAs in lung cells in response to noxious stimuli potentially suggest the originated cells of EV-cargo circRNAs.

### 3.4 Potential miRNAs predicted to interact with the designated circRNAs

One of the known functions of circRNA is to sponge regulatory non-coding RNAs, such as miRNAs (31). Using currently available tools (31), we predicted the miRNAs that potentially interact with the above sample circRNAs. As shown in **Supplementary Figure 2**, the miRNAs potentially interacting with circ06620, circ30884, and 25900 are listed. These predicted miRNAs potentially provide insights into the cellular functions of these circRNAs.

## 4 Discussion

In the past decades, circRNAs have been reported to mediate human diseases such as neurological diseases, cardiovascular diseases, and cancer (10–17). The expression, regulation, function, and mechanisms of circRNAs in the development of inflammatory lung responses have not been studied. To the best of our knowledge, this study is the first report showing that the circRNA profiles in BALF EVs were dramatically altered after both infectious and sterile stimuli. We further showed that in response to bacteria-derivatives, e.g. LPS, certain circRNAs were significantly altered in the BALF inflammatory cells and macrophages. The second significance of this study is that in addition to BALF-EV circRNA arrays, specific circRNAs can be validated using RT-qPCR using specific primers for the circular form. Given that circRNAs have much longer half-life due to the lack of 5’ or 3’ ends and resistance to exonuclease-mediated degradation, one could hypothesize that the EV-cargo circRNAs detected in BALF and serum potentially serve as biological markers reflecting the activation of immune cells and the inflammatory lung response. The third significance of this work is that circRNAs profiles also altered significantly between the sterile or non-infectious stimuli, e.g. acid instillations. This observation indicated that circRNAs may play crucial effects in the inflammatory process in response to all types of noxious stimuli. Furthermore, we also predicted the miRNAs potentially interacting with several specific circRNAs. This report is mainly to illustrate that circRNA profiles were altered significantly in the BALF in the pathogenesis of lung inflammation. CircRNA profiles altered regardless of the stimuli suggest that circRNAs may regulate certain central processes.

In this report, we validated the methods to study circRNAs in lung EVs and cells. We first used the high-throughput RNA-sequencing microarray screening by Arraystar. We used the microarrays rather than deep RNA-sequencing given that this is the first screening assay of circRNAs in mouse lung inflammation and the consideration of cost-effectiveness. It certainly has drawbacks, given that some alternative splicing and novel circRNAs may be missed using the existing microarray methods. However, the sequencing method also has difficulties in detecting the low abundance of circRNAs (32, 33). Since circRNAs have not been explored in mouse lung inflammation, we would examine the known candidates first. For each specific circRNA, high-throughput microarray data always requires validation. As shown in **Figure 2**, using RT-qPCR with specific primers for each circRNA, we validated several circRNAs from the screening using circRNA. As expected, some circRNAs cannot be validated using RT-qPCR. This is likely due to the low-abundance transcripts of these circRNAs. Additionally, as shown in **Supplementary Figure 1**, a time course may need to be performed to better characterize the expression of a specific circa, given that stimulation-induced circRNAs may be differentially detected at the different phases during the pathogenesis.

One of the key issues involving circRNA expression is that their linear host gene should be excluded. Primer crossing the BSJ region is designed and used in our report (**Figure 2A**). Notably, the BSJ-based method may not be able to detect or validate all known circRNAs. The field of circRNA is relatively new and the detection of circRNA expression using a primer crossing the BSJ region remains largely dependent on the full-length sequence of circRNA. The main method to determine the full-length sequence of circRANs is to simply combine all known mRNA exons in sequential order as putative full-length circRNA (1, 34). This method assumes that circular and linear transcripts share the same compositions. Other methods to determine the full-length sequences of circRNAs (2, 35, 36) require to identification of the internal components of the BSJ. This approach has a drawback in that only a small fraction of circRNAs can be identified by assembling BSJ reads (37). Therefore, reconstructing full-length circRNAs and quantifying circular isoforms are crucial steps for analyzing the circRNA isoform expressions, particularly to those circRNAs with low-abundance transcripts. Currently, several novel methods have been developed to predict the circRNA full-length sequences (38). We expected that with the increasing number of novel methods appearing in circRNA research, those circRNAs that are not validated currently require a re-evaluation.

CircRNA studies are now shifting from the identification of novel circRNAs to the determination of their functions, and to an extended level, their potential applications used in human health. CircRNAs potentially serve as an excellent novel target to develop a biomarker for certain human diseases thanks to their longer half-life and higher stability. However, the longer half-life of circRNAs may also create problems that reflect rapid biological processes. Therefore, detailed time courses are needed for the functional studies on each specific circRNA. Next, circRNAs are potential regulators of protein and other non-coding RNAs, adding another tier of regulation in cellular events. Indeed, one of the well-reported biological functions of circRNAs is miRNA sponging (31). Using the currently available bioinformatical tools, we predicted the potential miRNAs interacting with the selected circRNAs (**Supplementary Figure 2**). Again, all these candidate miRNAs require validations. Currently, multiple computational algorithms have been developed (31). One of the classic models to predict the circRNA-miRNA interaction was reported by Memczak et al. in 2013 (39). In recent years, accumulating reports confirmed that circRNAs carry the sponging effects of miRNAs via the competitive interactions with miRNAs, subsequently regulating the downstream proteins (40, 41). Novel models to predict the circRNA–miRNA interaction are emerging too, such as KGDCMI (42). Besides indirect regulation of downstream proteins, CircRNA potentially interacts with proteins directly and modulates their functions.

One area that remains largely unclear is circRNA elimination. Given their longer half-life and more resistance to degradation, presumably, circRNAs would accumulate intracellularly and trigger detrimental consequences. Recent reports suggest that the secretion out to extracellular space potentially serves as one of the methods to remove intracellular circRNAs. Our reports, for the first time, in BALF, studied the secretion of circRNAs via exosomes and analyzed the EV-cargo circRNA profiles, in the setting of non-infectious and endotoxin stimulation. Our studies suggest that the secretion of circRNAs via EVs is not a random process but may be stimuli dependent. For example, the profiles of circRNAs in BALF EVs after endotoxin were altered compared to those after non-infectious stimuli. This result potentially suggested that EV-cargo circRNAs may be developed into a marker reflecting cellular events in the process of human diseases. Furthermore, EV-cargo circRNAs may serve as concentrators of the “waste” circRNAs and facilitate them to be degraded in macrophages. EV is known to be taken into the cells and connected to the endosome/lysosome systems (43). Therefore, EV potentially serves as a “collector” or “concentrator” to gather all the intracellular “waste” circRNAs and transport them to the designated place (macrophage, etc) to be degraded.

There are several shortcomings in this report. First, using the microarray-based method will only explore known circRNAs, but may miss undiscovered ones. However, the sequencing method is not adequate for circRNA profiling due to the following reasons: Besides high costs, most circRNAs cannot be quantified due to low circular junction read counts. Furthermore, limited annotation information from the public circular RNA databases adds more difficulties to the reliability, accuracy, and computational methodology (36, 37). Second, some of the circRNAs may not be conserved in humans. Future directions include profiling the circRNAs using human specimens. A large-scale and systemic analysis using human specimens remains to be performed. Certainly, the biggest future direction will be functional analysis of each circRNA and/or a group of circRNAs.

In summary, our reports first investigated the EV-cargo circRNA profiles in BALF obtained after bacterial endotoxin or acid instillation in mice. We next confirmed our observations using RT-qPCR in EVs, lung tissue, and BALF cells. The detection of circRNAs requires specific primers that do not detect its linear form. CircRNAs may serve as a novel target in future research on inflammatory lung responses.

## Data availability statement

The original contributions presented in the study are publicly available. This data can be found here: https://figshare.com/s/f530ef56d3e54244115c.

## Ethics statement

Ethical approval was not required for the studies on humans in accordance with the local legislation and institutional requirements because only commercially available established cell lines were used. The animal study was approved by Institutional Animal Care & Use Committee - Boston University. The study was conducted in accordance with the local legislation and institutional requirements.

## Author contributions

HL: Writing – review & editing, Validation, Software, Methodology, Investigation, Formal analysis, Data curation. RH: Writing – original draft, Software, Methodology, Investigation, Formal analysis, Data curation. YJ: Writing – review & editing, Writing – original draft, Supervision, Resources, Project administration, Funding acquisition, Conceptualization.

## Supplementary material

Supplementary Figure 1 Evaluation of circRNAs in macrophages, lung tissue, and epithelial cells. **(A)** Left panels: WT mice were exposed to LPS (1µg/mouse) and after 24h, mouse lung tissue was homogenized, and RNA was isolated. The designated circRNAs were analyzed using the same primers described above by RTqPCR. **(B)** Right panels: Mouse lung alveolar type I cell line (E-10) cells were cultured *in vitro* as previously described (44). After exposure to LPS (100 ng/ml, 24h, top panels), RNA was isolated from these cells and subjected to RT-qPCR. For the lower panels, immortalized AMs were treated with LPS (100ng/ml) for a time course. Circ25900 expression was analyzed by RT-qPCR. The figures shown here represent repeats from two independent experiments. *, ** p<0.05.

Supplementary Figure 2 Prediction of miRNAs that interact with the designated circRNAs. We selected the three circRNAs detectable in BALF cells as an example. The predicted miRNAs that potentially interacted with the designated circRNAs are listed. The left columns show the potential miRNAs binding with the specific circRNAs. The right columns indicate the number of predicted binding sites on these circRNAs.
