# Supplementary figures and images for: Altered circular RNA expressions in extracellular vesicles from bronchoalveolar lavage fluids in mice after bacterial infections
Source: Front Immunol. 2024 Apr 4;15:1354676. doi: 10.3389/fimmu.2024.1354676 (PMC11024224; doi:10.3389/fimmu.2024.1354676)

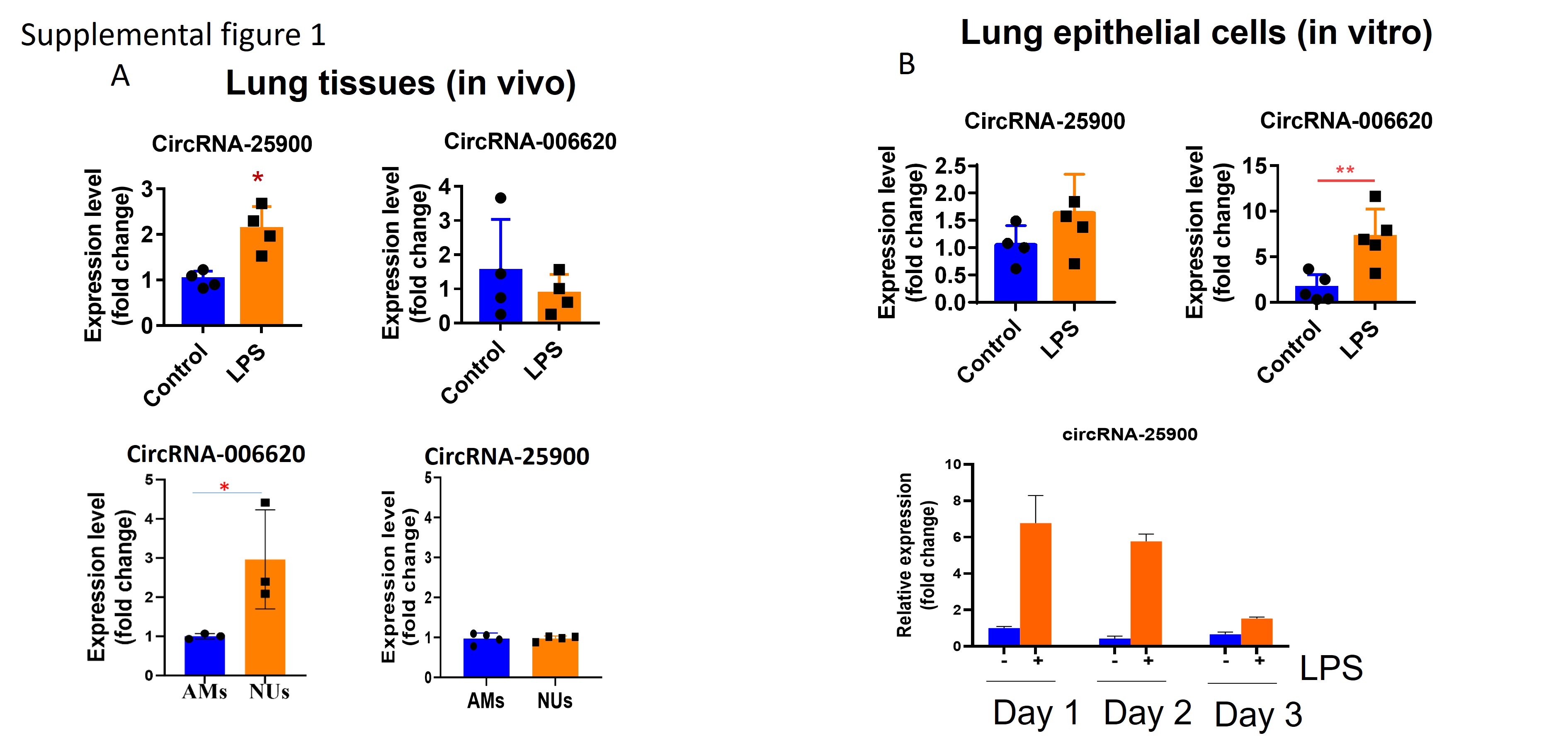

Supplement: Supplementary Figure 1 — Evaluation of circRNAs in macrophages, lung tissue, and epithelial cells. (A) Left panels: WT mice were exposed to LPS (1µg/mouse) and after 24h, mouse lung tissue was homogenized, and RNA was isolated. The designated circRNAs were analyzed using the same primers described above by RTqPCR. (B) Right panels: Mouse lung alveolar type I cell line (E-10) cells were cultured in vitro as previously described (44). After exposure to LPS (100 ng/ml, 24h, top panels), RNA was isolated from these cells and subjected to RT-qPCR. For the lower panels, immortalized AMs were treated with LPS (100ng/ml) for a time course. Circ25900 expression was analyzed by RT-qPCR. The figures shown here represent repeats from two independent experiments. *, ** p<0.05. [file Image_1.jpeg]

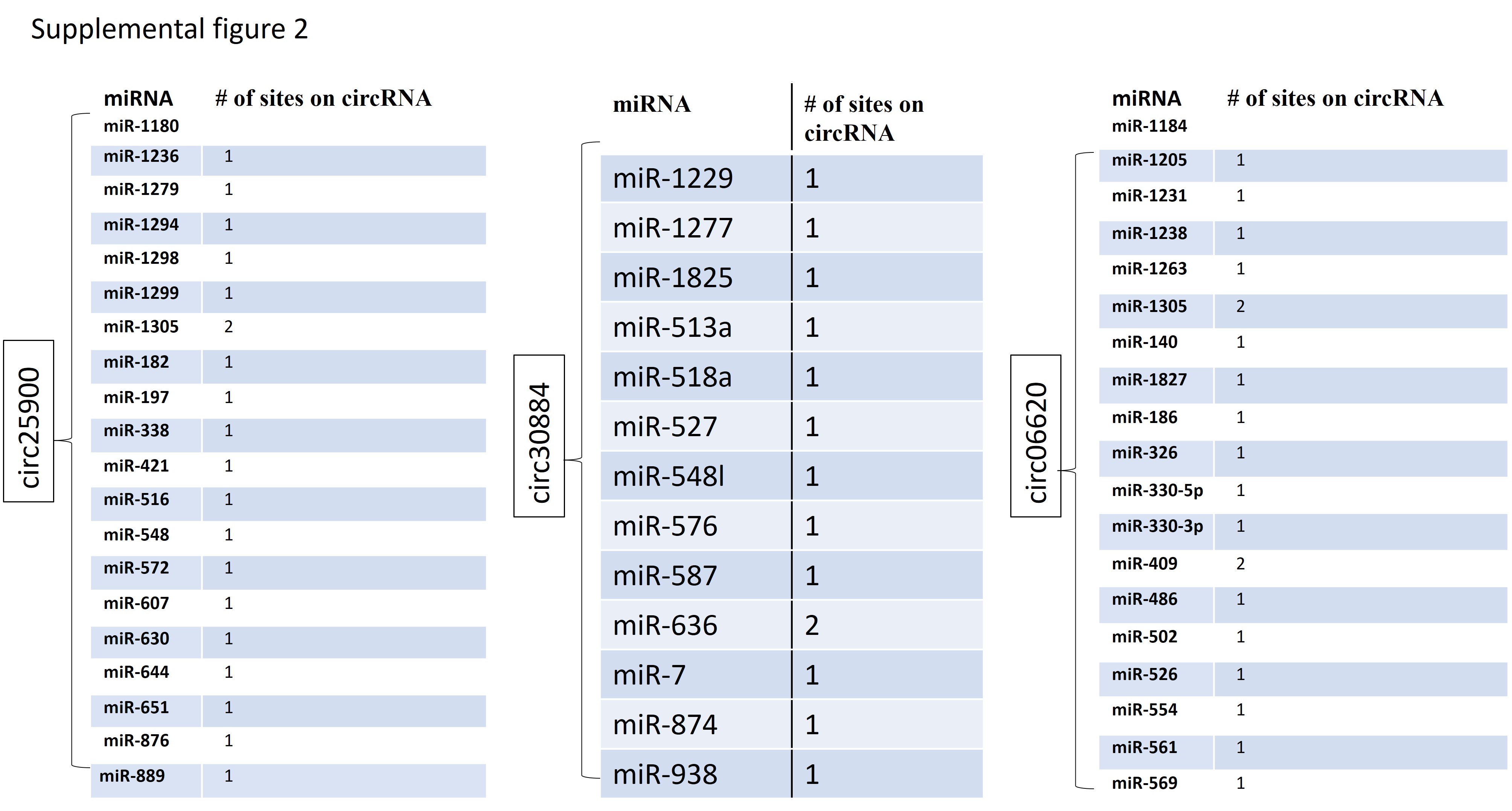

Supplement: Supplementary Figure 2 — Prediction of miRNAs that interact with the designated circRNAs. We selected the three circRNAs detectable in BALF cells as an example. The predicted miRNAs that potentially interacted with the designated circRNAs are listed. The left columns show the potential miRNAs binding with the specific circRNAs. The right columns indicate the number of predicted binding sites on these circRNAs. [file Image_2.jpeg]
